
JOURNAL INFORMATION
==============================
NLM Title Abbreviation: Am J Trop Med Hyg
Journal ID: Am J Trop Med Hyg
NLM Journal ID: 0370507
Journal ID: tpmd

The American Journal of Tropical Medicine and Hygiene

ISSN: 0002-9637
EISSN: 1476-1645
Publisher: The American Society of Tropical Medicine and Hygiene

ARTICLE INFORMATION
==============================
PMCID: PMC11448487
DOI: 10.4269/ajtmh.1114stoc
Article ID: tpmd1114stoc
Article version: 1
Subjects: Other

Contents

Electronic publication date: 2024 Oct 1
Print publication date: 2024 Oct
Volume: 111
Issue: 4 Suppl
Issue title: Clinical Trials to Assess Effectiveness of Treatment Guidelines for Lymphedema Induced by Lymphatic Filariasis
First page: ii
Last page: ii
Copyright: © 2024, The American Society of Tropical Medicine and Hygiene
Copyright year: 2024
License: This is an open-access article distributed under the terms of the Creative Commons Attribution (CC-BY) License, which permits unrestricted use, distribution, and reproduction in any medium, provided the original author and source are credited.
License URL: https://creativecommons.org/licenses/by/4.0/

Page count: 1

==============================

### Clinical Trials to Assess Effectiveness of Treatment Guidelines for Lymphedema Induced by Lymphatic Filariasis

### Guest Editor: James W. Kazura

### CONTENTS

**PART 1**
**The Biology and Strategies for Managing Filarial Lymphedema**

**EDITORIAL**

**Narrowing the Gap in Managing the Lymphedema of Lymphatic Filariasis**
James W. Kazura 1

**REVIEW**

**Managing Lymphedema Induced by Lymphatic Filariasis: Implementing and Improving Care at the Individual and Programmatic Levels**
Charles D. Mackenzie, D Ramaiah Kapa, Suma Krishnasastry, Jan Douglass, Achim Hoerauf, and Eric A. Ottesen 3

**PART 2**
**Clinical Trials for Treating and Managing Filarial Lymphedema**

**Effect of Adding a Six-Week Course of Doxycycline to Intensive Hygiene-Based Care for Improving Lymphedema in a Rural Setting of Mali: A Double-Blind, Randomized Controlled 24-Month Trial**
Yaya I. Coulibaly, Abdoul F. Diabate, Moussa Sangare, Sekou O. Thera, Housseini Dolo, Salif S. Doumbia, Siaka Y. Coulibaly, Ayouba Diarra, Lamine Diarra, Diadje Tanapo, Michel E. Coulibaly, Lamine Soumaoro, Abdallah A. Diallo, Amatigue Zeguime, Yacouba Sanogo, Adama Berthe, Fatoumata Dite Nene Konipo, Charles Mackenzie, Mariana Stephens, Joseph P. Shott, Jayla Norman, Ute Klarmann-Schulz, Achim Hoerauf, Andrew Majewski, John Horton, Sarah Sullivan, Eric A. Ottesen, and Thomas B. Nutman 22

**Efficacy of Intensified Hygiene Measures with or without the Addition of Doxycycline in the Management of Filarial Lymphedema: A Randomized Double-Blind, Placebo-Controlled Clinical Trial in Tanzania**
Abdallah Ngenya, Ute Klarmann-Schulz, Winfrida John, Patricia Jebett Korir, Mathias Kamugisha, Jennifer Nadal, Dennis Moshi, Arcangelo Ricchiuto, Ndekya Oriyo, Sarah Mary Sullivan, Ruth Laizer, John Horton, Max Demitrius, Anja Feichtner, Thomas F. Marandu, Yusuph Mgaya, Angelika Kellings, Inge Kroidl, John Ogondiek, Janina M. Kuehlwein, Leonard Masagati, Charles Mackenzie, Maureen Mosoba, Sacha Horn, Kheri Kagya, Samuel Wanji, Wilfred Mandara, Linda Batsa Debrah, Eric A. Ottesen, Alexander Yaw Debrah, Upendo Mwingira, Achim Hoerauf, and Akili Kalinga 33

**LEDoxy-SL: A Placebo-Controlled, Double-Blind, Randomized, 24-Month Trial of Six Weeks of Daily Doxycycline Plus Hygiene-Based Essential Care for Reducing Progression of Filarial Lymphedema in Sri Lanka**
Thishan Channa Yahathugoda, Nirmitha Lalindi De Silva, Janaka Ruben, Sharmini Gunawardena, Mirani Vasanthamala Weerasooriya, John Horton, Philip Budge, Eric Ottesen, Sarah Mary Sullivan, Mariana Stephens, John Shen, Ute Klarmann-Schulz, Achim Hoerauf, Joseph Patrick Shott, and Charles Mackenzie 52

**Adherence to Hygiene Protocols and Doxycycline Therapy in Ameliorating Lymphatic Filariasis Morbidity in an Endemic Area Post-Interruption of Disease Transmission in Ghana**
Linda Batsa Debrah, Ute Klarmann-Schulz, Jubin Osei-Mensah, Janina M. Kuehlwein, Yusif Mubarik, Jennifer Nadal, Nana Kwame Ayisi-Boateng, Arcangelo Ricchiuto, Vera Serwaa Opoku, Sarah M. Sullivan, Derrick Adu Mensah, John Horton, Abu Abudu Rahamani, Philip J. Budge, Stephen Gbedema, Patricia Jebett Korir, John Opoku, Kenneth Pfarr, Derrick Boateng Kontoh, Angelika Kellings, Charles Gyasi, Michael Agyemang Obeng, Barbara Gruetzmacher, Fatima Amponsah Fordjour, Inge Kroidl, Sacha Horn, Eunice Kyaakyile Kuutiero, Caroline Wauschkuhn, Abdallah Ngenya, Charles Mackenzie, Samuel Wanji, Akili Kalinga, Eric A. Ottesen, Achim Hoerauf, and Alexander Yaw Debrah 66

**Efficacy and Safety of Adding 6 Weeks of Doxycycline to the Essential Package of Care to Treat Filarial Lymphedema: A Double-Blind, Randomized, Controlled Trial in Southern India**
Suma Krishnasastry, Anuja Ashok, Ammu Devidas, Sarah Sullivan, Mariana Stephens, Jayla Norman, Elianna Paljug, Andrew Deathe, Andrew Majewski, John Horton, Joseph P. Shott, Ute Klarmann-Schultz, Achim Hoerauf, Eric Ottesen, and Charles D. Mackenzie 83

For more information about
the American Society of Tropical Medicine and Hygiene
please visit us at www.astmh.org

The American Journal of Tropical Medicine and Hygiene is
online at www.ajtmh.org
